# Rationale and design of the EPCHF trial: the early palliative care in heart failure trial (EPCHF)

**DOI:** 10.1007/s00392-021-01903-1

**Published:** 2021-07-09

**Authors:** Marc Ulrich Becher, Mahmoud Balata, Michaela Hesse, Fabian Draht, Christian Zachoval, Birgitta Weltermann, Ralf Westenfeld, Martin Neukirchen, Roman Pfister, Thomas Standl, Lukas Radbruch, Georg Nickenig

**Affiliations:** 1 Department of Cardiology, Angiology, Pneumology and Medical Intensive Care, University Hospital Bonn, Venusberg-Campus 1, 53127 Bonn, Germany; 2 Department of Palliative Medicine, University Hospital Bonn, Bonn, Germany; 3 Institute of General Practice and Family Medicine, University Hospital Bonn, Bonn, Germany; 4 Department of Cardiology, University Hospital Düsseldorf, Düsseldorf, Germany; 5 Interdisciplinary Centre for Palliative Care, University Hospital Düsseldorf, Düsseldorf, Germany; 6 Department of Cardiology, University Hospital Cologne, Cologne, Germany; 7 Department of Anesthesia, Intensive Care and Palliative Medicine, Städtisches Klinikum Solingen, Solingen, Germany

**Keywords:** Palliative care, Heart failure, Quality of life, FACIT-PAL, KCCQ

## Abstract

The progressive nature of heart failure (HF) coupled with high mortality and poor quality-of-life (QoL) mandates greater attention to palliative care (PC) as a routine component of HF management. Limited evidence exists from randomized controlled trials supporting the use of interdisciplinary palliative care in the progressive course of HF. The early palliative care in heart failure trial (EPCHF) is a prospective, controlled, nonblinded, multicenter study of an interdisciplinary palliative care intervention in 200 patients with symptomatic HF characterized by NYHA ≥ 2. The 12-month EPCHF intervention includes monthly consultations by a palliative care team focusing on physical and psychosocial symptom relief, attention to spiritual concerns and advance care planning. The primary endpoint is evaluated by health-related QoL questionnaires after 12 months of treatment. First the functional assessment of chronic illness therapy palliative care (FACIT-Pal) score evaluating QoL living with a chronic disease and second the Kansas City cardiomyopathy questionnaire (KCCQ) measuring QoL living with heart failure will be determined. Secondary endpoints are changes in anxiety/depression (HADS), symptom burden score (MIDOS), spiritual well-being functional assessment of chronic illness therapy spiritual well-being scale (FACIT-Sp), medical resource and cost assessment. EPCHF will help evaluate the efficacy and cost-effectiveness of palliative care in symptomatic HF using a patient-centered outcome as well as clinical and economic endpoints. EPCHF is funded by the Bundesministerium für Bildung und Forschung (BMBF, 01GY17).

## Background and rationale

The epidemiological burden of heart failure (HF) is tremendous, with an estimated 26 million people affected worldwide [1]. HF currently affects over 2 million patients in Germany. Accordingly, HF was the most common cause of disease-related hospitalization, the second leading cause of hospital stay, and the leading cause of in-hospital deaths in Germany in 2013 [1]. Despite recent therapeutic advances, HF is a debilitating syndrome that results in a high burden of symptoms and poor QoL. Patients suffer not only from physical effects but also from psychosocial and spiritual distress [2, 3]. Selected HF patients are candidates for aggressive treatments, such as cardiac transplantation or mechanical circulatory support; nevertheless, the application of these therapies to the broader HF population is limited by constrained resources [4]. The progressive nature of HF coupled with high mortality rates and poor QoL mandates greater attention to palliative care (PC) as a routine component of HF management [5].

PC with its focus on management of symptoms, psychosocial support, and assistance with decision making has the potential to improve the quality of care and reduce the use of medical services [3, 4]. However, PC has traditionally been delivered late in the course of disease to patients who are hospitalized in specialized inpatient units or as a consultative service for patients with uncontrolled symptoms [5, 6]. Previous studies have suggested that late referrals to palliative care are inadequate to alter the quality and delivery of care provided to patients [7, 8]. To have a meaningful effect on patients’ QoL and end-of-life care, PC services should be provided earlier in the course of the disease. We here postulate that introducing of PC earlier is feasible and beneficial among patients with symptomatic HF in the outpatient setting.

PC is a multidisciplinary approach that focuses on providing patients with relief from symptoms, pain, and stress of living with a serious illness at any stage of disease [6]. The goal of the current study is to examine the effect of early integration of PC within standard cardiac care on QoL, reported cardiac outcomes, and the use of health services among HF patients.

We hypothesized that patients who received early PC in the ambulatory care setting, as compared with patients who received standard cardiac care alone, would have a better QoL due to lower rates of depressive symptoms, symptom burden, unmet spiritual needs, reported cardiac outcomes and less aggressive end-of-life care thereby improving QoL for both, patients and their caregivers.

Despite consensus-panel and guideline recommendations the integration of PC with evidence-based HF therapies in the later stages of disease is limited [9, 10]. Many physicians equate PC with end-of-life care and prognostic uncertainty poses a challenge in choosing the appropriate time point to implement PC. Furthermore, cardiologists typically lack formalized training in the PC-principles and it is less clear which HF patients benefit from palliative interventions and which interventions improve QoL achieving outcomes desired by patients and family members [9]. However, multiple recent pilot studies in HF populations have suggested that PC may reduce symptom burden and improve QoL [11–13]. These studies have highlighted the importance of symptoms, such as anxiety and depression in HF patients in addition to the commonly recognized symptoms of fatigue, dyspnea and nausea. Moreover, a randomized trial of PC in Stage 4 lung cancer showed improved patient’s symptoms, improves QoL and patients randomized to PC survived longer than those randomized to usual care [14]. Furthermore, multiple studies in oncology and noncancer illness patients have shown reduced costs leading to increased interest of whether similar results can be found in other common diseases, such as HF [15–17]. These studies provide the foundation and rationale for a large-scale randomized HF trial sufficiently powered to assess the effect of early palliative care on clinical outcomes in HF patients.

To help create this body of evidence, the BMBF has funded the early palliative care in heart failure trial (EPCHF) to evaluate if a multidimensional palliative care intervention improves health-related outcomes relative to usual care alone in symptomatic HF patients (Registration No.: DRKS Register 00013922, Studiencode: MED2-201604_EPCHF, Sponsor: BMBF FKZ: 01GY1704). This article describes the design and rationale of the EPCHF trial (https://www.gesundheitsforschung-bmbf.de/de/epchf-studie-fruhe-palliativmedizinische-intervention-bei-patientinnen-und-patienten-mit-7630.php). The authors are solely responsible for the design and conduct of this study, the drafting and editing of this paper and its final contents. Of note, the BMBF is currently funding multiple clinical trials and research projects related to palliative care (see https://www.bmbf.de).

## Methods

### Trial design

**Fig. 1:**
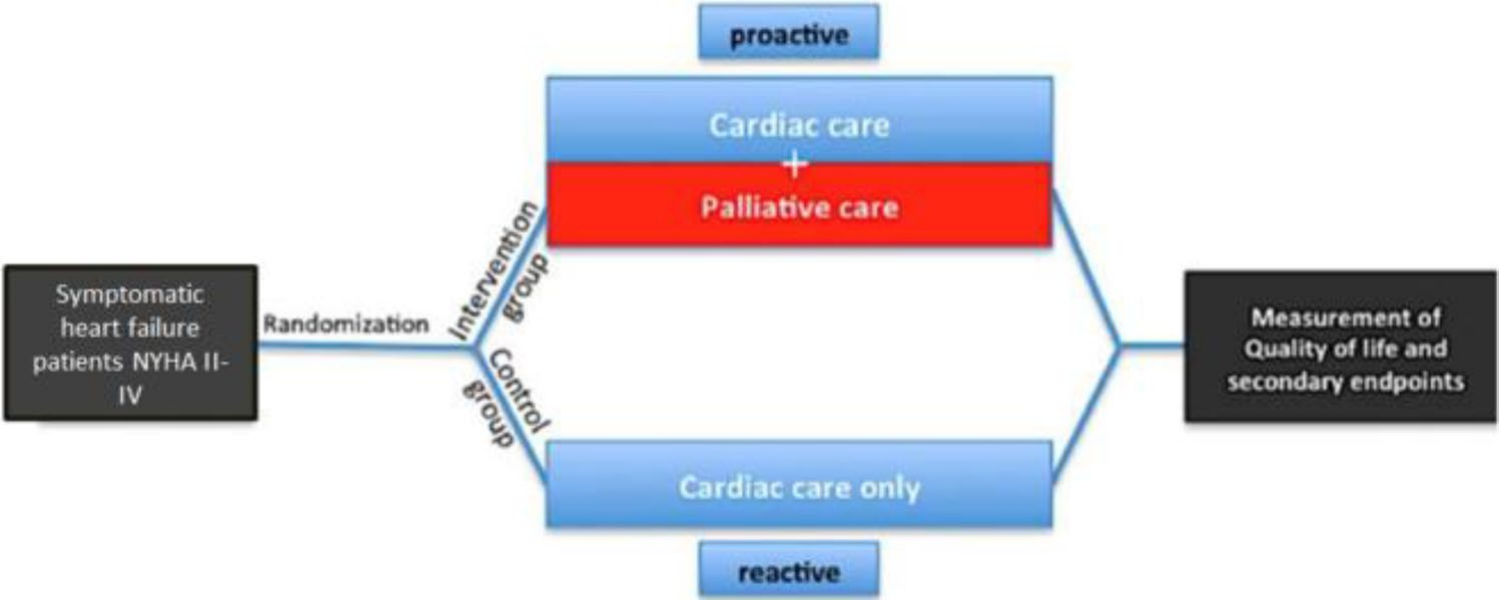
Intervention scheme of EPCHF. Experimental intervention: additional palliative care to standard cardiac care. The intervention includes monthly consultations by a palliative care team over 12 months (palliative care assessment of physical, psychological, social and spiritual needs, establishing goals of care, assisting with decision making) in the outpatient or inpatient setting. Control intervention: standard cardiac care with palliative care on demand only. Duration of intervention per patient: 12 months. Time of data collection: at baseline and at 4 follow-up visits every 3 months within 12 months

**Fig. 2:**
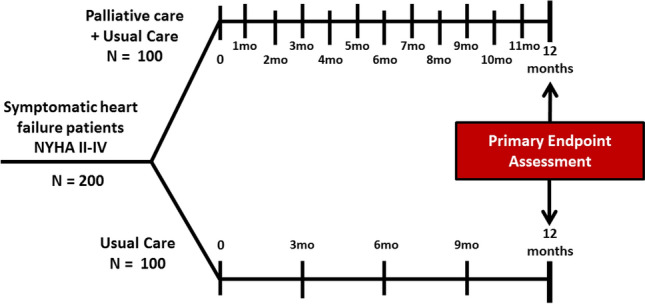
Trial flow of EPCHF. The individual treatment duration is 12 months. Trial duration: recruiting period: 26 months. Planned start date (FPFV): 01.04.2018. Planned end date (LPLV): 31.07.2021. Time for preparation of the trial (months): 3 months. First patient in to last patient out (months): 38 months. Time for data clearance and analysis (months): 3 months. Duration of the entire trial (months): 42 months

**Fig. 3:**
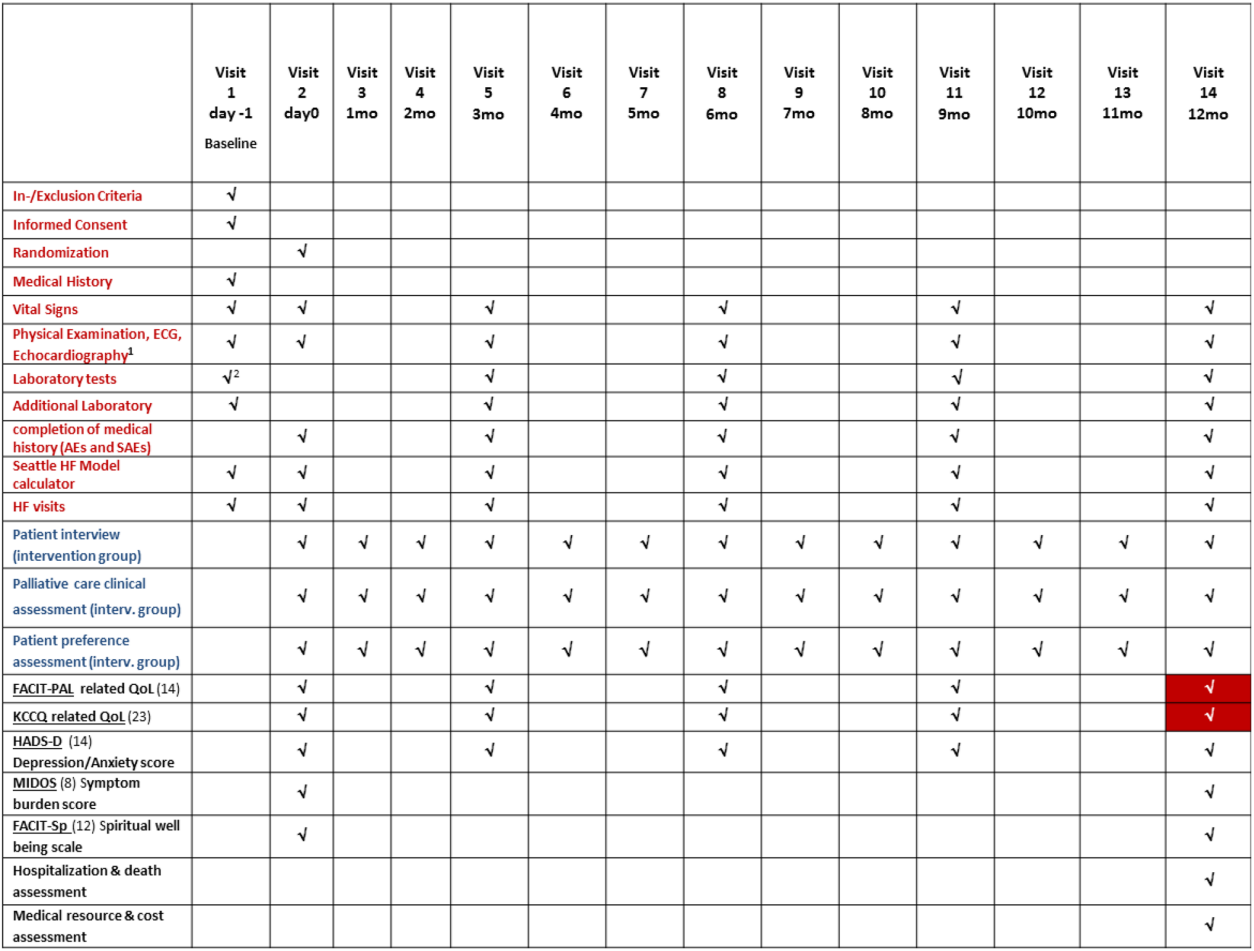
Schedule of activities. Red font: best supportive cardiac care (BSCC). Blue font: additional early integration of palliative care (EIPC). Black font: conducted investigations. ^1^LVEF, LV diastolic dysfunction, hemodynamic, ^2^incl. negative blood pregnancy test for women of childbearing potential

EPCHF is a prospective, controlled, two-arm, non-blinded, randomized, multicenter clinical trial of early palliative care integrated with standard cardiac care, as compared with standard cardiac care alone (Fig. 1). The multicenter study will be performed at the departments of Internal Medicine and Palliative Care of the University Hospital of Bonn and departments of Internal Medicine and Palliative Care of the University Hospital of Düsseldorf. Eligible patients with symptomatic HF and dyspnea NYHA ≥ 2 will be randomly assigned to one of the two groups in a 1:1 ratio to usual contemporary HF care or usual care combined with the EPCHF intervention (Fig. 1). Subjects are assigned treatment using a complete randomization scheme. Patients who are assigned to early PC will meet with a member of the PC team, which consisted of board-certified PC physicians and advanced-practice nurses within the time of hospitalization after enrollment and at least monthly thereafter in the outpatient setting during the 12 months of follow-up (Figs. 2 and 3). The palliative care intervention focuses on symptom relief, attention to spiritual concerns, and advance care planning. The trial is non-blinded, since it is not possible to execute a double-blinded trial of the EPCHF intervention given that the intervention involves a multidisciplinary team. The duration of the intervention is 12 months, but patients in both groups are followed until death or the end of the study (~ 3.5 years). The primary endpoint refers to health-related QoL after 12-month follow-up (Fig. 3).

### Study population

The study will enroll patients with symptomatic HF defined by dyspnea NYHA ≥ 2. Inclusion and exclusion criteria are presented in Tables 1 and 2. Mortality risk due to HF will be calculated by the Seattle Heart Failure Score [18].

**Table 1:** Inclusion criteria

Inclusion criteria	Subjects will only be included in the study if they meet all of the following criteria
	Age ≥ 18 years
	NYHA class II–IV symptoms (suspected cardiac dyspnea)
	Systolic HF with a LVEF ≤ 35% or HF with preserved ejection fraction (NYHA class II–IV, elevated natriuretic peptides (NP), left ventricular hypertrophy (LVH) or left atrial enlargement (LAE) or diastolic dysfunction
	BNP ≥ 100 pg/mL or NT proBNP ≥ 400 pg/mL
	Subjects with the ability to follow study instructions and likely to attend and complete all required visits
	Written informed consent

**Table 2:** Exclusion criteria

Exclusion criteria	Subjects will not be included in the study if any of the following criteria applies
	Inability to read, understand and respond to question in German
	Previous consultations of palliative care services
	Patients in intensive care unit, on a ventilator, pre- or post-heart transplant
	Non-cardiac terminal illness
	Women who are pregnant or planning to become pregnant, nursing women
	Simultaneously participation in another study or participation in any study involving administration of an investigational medicinal product within 30 days prior to study beginning
	Subjects with a physical or psychiatric condition which at the investigator’s discretion may put the subject at risk, may confound the trial results, or may interfere with the subject’s participation in the study
	Known or persistent abuser of medication, drugs or alcohol

### Usual care

Patients are managed by a cardiologist-directed team with HF expertise. HF patient care is focused on symptom relief and use of evidence-based therapies [19, 20]. Additional goals of care include treatment of co-morbidities and patient education designed to assist with self-management techniques. For instance, we routinely target sleep disordered breathing and mood disorders, and also encourage exercise training in our HF patients as recommended by guidelines [20]. For ethical reasons, inpatient palliative care consultation at the discretion of the attending cardiologist is not denied to Usual Care patients. HF patients generally receive outpatient follow-up with an HF cardiologist focused on guideline-based medication titration, assessment of adherence to medical and dietary regimens, and serial monitoring of end-organ function. The outpatient palliative care departments of all participating centers are based not readily available to Usual Care patients. As the value of outpatient PC in the HF population is undefined, we believe it is ethically acceptable for outpatients in this arm to receive only state-of-the-art usual cardiovascular care.

### Palliative care intervention

An interdisciplinary, guideline-driven, multi-component PC intervention has been designed and administered with contemporary HF management. The goal is a structured and reproducible approach to assessing and managing the multiple domains of QoL for patients with advanced HF including physical symptoms, psychosocial concerns, spiritual concerns, and advance care planning. At the core of the palliative intervention team is a PC trained NP who coordinates this aspect of the patient’s care, a palliative care board-certified physician, and a trained counselor. Since the same cardiology team cares for patients in both the intervention and control group, the palliative care intervention is performed in collaboration with the cardiology team, but does not involve specific cardiology-based palliative interventions. This approach was designed in an attempt to minimize the extension of PC-specific interventions into the usual care study group to maintain integrity of the study design. Specific attention will be paid to assessing physical and psychosocial symptoms, establishing goals of care, assisting with decision making regarding treatment, and coordinating care on the basis of the individual needs of the patient [21].

### Physical symptoms

At study enrollment and pre-specified time points, the NP performs standardized assessments to determine the presence of commonly experienced symptoms, including dyspnea, fatigue, pain, anxiety, depression, nausea, anorexia, constipation, and insomnia. Symptoms are managed by the EPCHF palliative team using treatment algorithms to ensure standardization. In addition to periodic assessment and protocolized management of symptoms, patients in both arms are provided with an *EPCHF heart failure action plan* handout with instruction on regular control of signs that HF is getting worse and what to do if these changes occur. The handout includes information on self-management of symptoms associated with congestion and on medications typically used for HF on “as needed” basis for symptom relief at home, such as loop diuretics, and sublingual nitroglycerin [6, 22]. Patients of the interventional arm receive a handout including potential medications that may be prescribed for managing anxiety, nausea, vomiting and constipation. The medications themselves are prescribed at the discretion of the EPCHF clinical provider.

### Psychosocial symptoms

Patients are screened for symptoms of anxiety and depression using the hospital anxiety and depression scale (HADS) [23]. The HADS has been widely used in clinical trials as a psychological screening test for the states of anxiety and depression [24]. Patients screened positive for either anxiety or depression receive more thorough assessment and management as specified by a treatment algorithm used to determine the need for a mental health referral and the possible use of antidepressants, anxiolytics, stress management techniques, and psychotherapy.

### Spiritual concerns

The EPCHF NP completes a spiritual assessment at the time of patient enrollment or at the first outpatient visit after hospitalization using the FICA Spiritual History Tool [25]. FICA is an acronym which serves as a guide and includes: F—Faith and Beliefs; I—Importance in life and health; C—Community–Religious and Spiritual Community; A—How the patient would like spirituality addressed in medical care. The NP shares information gathered in the spiritual assessment with the palliative team and addresses specific spiritual concerns as appropriate. If needed, the NP arranges subsequent visits addressing concerns raised during the initial assessment.

### End-of-life preparation

To address end-of-life preparation, we use the Outlook Intervention which includes three 1-h sessions, spaced 1 week apart [26]. Session 1 focuses on life review, accomplishments, proudest moments, and cherished times. Session 2 focuses on issues of forgiveness, things the patient would have done differently, and things left unsaid or undone. Session 3 focuses on lessons learned, heritage, and legacy. To ensure replicability, the intervention follows questions outlined in the Outlook Intervention training manual. The Outlook Intervention is administered by the trained counselor.

### Goals of care

To ensure that the palliative care intervention is specified and replicable, we followed a strict fundamental protocol. Every patient randomized to palliative care was contacted every 28 days with 5-day range of tolerance. The palliative care round was structured by the themes of the modified HOPE questionnaire [27]. Patients in need for help, e.g., with applications have additional appointments by phone or in attendance. In case of symptom management e. g. pain, we contact the cardiologists or GP by phone and gave a recommendation or we involve the pain-OPD to provide prescriptions. Further palliative medical support is highly individual and depends on wishes/topics set by the patient during conversation. The intervention includes communication designed to elicit patients’ goals of care which means to determine what is most important to individuals and use those goals to frame discussions of prognosis and of life-prolonging therapies. These conversations are facilitated by the NP and revisited periodically to document changes in preferences. To ensure standardization of PC treatment, the NP was trained in a 2-day intensive workshop based on the palliative care student’s curriculum, originally designed to provide medical students with communication skills to facilitate discussions with seriously ill patients [28]. To assess this aspect of the PC intervention, we document code status, completion of advance directives, and preferences for life-prolonging therapies.

After the 12-month intervention period is completed, the NP continues to contact the patients in the intervention arm every 3 months to provide ongoing support and clinical care. In addition, the HF team is available for phone consultation to patients randomized to the intervention arm throughout the duration of the trial.

### Measurements

The primary end point is evaluated by health-related quality-of-life questionnaires after 12 months of treatment (Fig. 3). First, the functional assessment of chronic illness therapy palliative care (FACIT-Pal) score evaluating QoL living with a chronic disease and second the Kansas City Cardiomyopathy Questionnaire (KCCQ) measuring QoL living with heart failure will be determined. The FACIT-Pal is a 14-item measure of self-reported QoL that assesses several domains including physical wellbeing, social/family well-being, emotional well-being, and functional well-being [29]. KCCQ is a 23-item, disease-specific questionnaire scored from 0 to 100 with higher scores representing better health status [30]. A 5-point change in the KCCQ overall summary score has been demonstrated to be the minimally detectable clinical difference [26, 28–34].

Secondary end points are changes in anxiety/depression (HADS), symptom burden score (MIDOS), spiritual well-being Functional Assessment of Chronic Illness Therapy Spiritual Well-Being Scale (FACIT-Sp), medical resource and cost assessment, and a composite of death, HF hospitalization, and quality of life.

### Measures of health care use

The evaluation of medical resources and costs is carried out using administrative data from the computer system (KAS Orbis) of the electronic medical record of the University Hospital Bonn to analyze costs and resource usage data. The amount paid for care (e. g. through compulsory or private health insurance) should be used as a primary cost estimate. In addition, sensitivity analyzes are performed using the estimated costs of care as a measure of resource use. Differences in the use of resources in the study arms are examined in order not only to determine the extent of the cost differences, but also to identify the sources that make the difference. At all visits, patients are asked if they have been treated outside the study hospital. If so, they are asked to estimate the number of visits and/or days in the hospital. The cost of such care is estimated on the basis of the medical expenditure survey and included in the total cost of care from the time of randomization until the completion of the study.

### Data collection

Participants complete baseline questionnaires before randomization. A complete schedule of assessments is given in Fig. 3. Follow-up evaluations of QoL and mood are expected to occur at 3 months, 6 months, 9 months and 12 months. As necessary, we will mail questionnaires to subjects and/or complete questionnaires over the telephone to help ensure the collection of primary endpoint data. When responses on questionnaires were incomplete, research staff documented the reasons for which the participant did not give a full response.

### Statistical analysis

Primary endpoints: the two primary endpoints will be tested in a two-step procedure. In the first step the hypothesis of equal scores for the FACIT-Pal in the two treatment groups will be tested with a two-side *t* test at a level of 5%. In case of the rejection of the null-hypothesis in the first step, the second hypothesis of equal KCCQ-scores in the two treatment groups will be tested again with a two-sided *t* test at the level of 5%. This test procedure controls a family wise error rate of 5%.

A comparably high number of drop-outs (~ 20%) especially due deaths are to be expected. Since this kind of censoring is definitely correlated to the QoL-measurement, the results for the QoL-score might be biased. An optimal choice of a procedure for the replacement of missing values would require a detailed knowledge about this correlation, which is not available. Missing values will not be replaced for the primary analysis of the two primary outcome variables. A sensitivity analysis will be performed replacing missing values by the last observation carried forward method.

For both variables the course of QoL scores over the observational time will analyzed descriptively by fitting mixed linear models to the data. 95%-confidence interval will be estimated for the score values separated by treatment groups and for measurement times. A second identical analysis for the primary endpoint will be performed based on the per-protocol population (PP), which is defined as the patients treated and observed according to protocol. This analysis will only be performed if ITT and PP-population differ.

Secondary endpoints: the secondary endpoints will be analyzed descriptively in the intention-to-treat collective.

Psychosocial symptoms: patients are screened for symptoms of anxiety and depression using the Hospital Anxiety and Depression Scale (HADS). The HADS has been widely used in studies as a psychologic screening test for the states of anxiety and depression. Patients who screen positive for either anxiety or depression receive a more thorough assessment and management as specified by a treatment algorithm used to determine the need for a mental health referral and the possible use of antidepressants, anxiolytics, stress management techniques, and psychotherapy.

Spiritual concerns: the EPCHF-HF NP completes a spiritual assessment at the time of patient enrolment or at the first outpatient visit after hospitalization using the FACIT-Sp. The NP shares information gathered in the spiritual assessment with the EPCHF team and addresses specific spiritual concerns as appropriate. If needed, the NP arranges for subsequent visits to address concerns raised during the initial assessment.

Moreover, qualitative interview data will supplement the results of questionnaires and gain information about the impact of gender issues and social inequality (e.g., patients with migrant background within the study). In addition, public health researcher with special expertise in the area of health inequality research will analyze the issue between over-indebtedness and HF. Because of the expected relevance of variables pertaining to socioeconomic status and migration background for HF-related outcomes including psychosocial aspects/issues, a careful evaluation of the associations between primary and secondary endpoints and these variables will be investigated.

The secondary endpoints HADS-D, MIDO, FACIT-Sp will be analyzed for differences between the treatment groups by fitting mixed linear models to the data. 95%-confidence interval will be estimated for the score values separated by treatment groups and for measurement times.

The survival times will be compared between the treatment groups by a log-rank test. Cox-proportional hazard models will be used to further explore the impact of the treatment on the survival of the patients.

The frequency of adverse events will be counted in absolute and relative frequencies, separated by treatment groups and depending on severity and relationship to the therapy.

Further details of the analyses for primary and secondary outcomes will be specified in a statistical analysis plan.

### Trial organization

A Data and Safety Monitoring Board (DSMB) has been appointed to review the conduct and results of this trial at 2 enrollment landmarks (after 33% and 66% of the patients have been enrolled). The DSMB is charged with reviewing safety composite data in both arms of the trial. The DSMB will be empowered to stop the study for evidence of harm, but not for evidence of lack of efficacy. The DSMB is also asked to offer perspective on any therapeutic or diagnostic testing advances that may occur during the trial course that may influence the outcome. If protocol modifications are warranted, close consultation among the DSMB and the study leadership will be required.

## Summary

HF creates significant physical, psychosocial and spiritual burden for patients and their families. Palliative care may represent an important component of the holistic management of patients with not only advanced HF. Further evidence from randomized trials of a palliative care intervention in HF is required to identify which patients may benefit from specific interventions. EPCHF aims to provide empiric data to support the efficacy and cost-effectiveness of palliative care improving the health-related QoL in HF patients after hospitalization. Such evidence could provide impetus to overcome current challenges related to limited access to, and adoption of, palliative care services for HF patients.

## Abbreviations

BMBF: Bundesministerium für Bildung und Forschung
BNP: B-type natriuretic peptide
EPCHF: Early palliative care in heart failure trial
FACIT-Pal: Functional assessment of chronic illness therapy palliative care
FACIT-Sp: Functional assessment of chronic illness therapy spiritual well-being scale
HF: Heart failure
KCCQ: Kansas City cardiomyopathy questionnaire
HADS: Hospital anxiety and depression scale
LVEF: Left ventricular ejection fraction
MIDOS: Minimal documentation system
NT-proBNP: N-terminal pro-B-type natriuretic peptide
NYHA: New York Heart Association
PC: Palliative care
QoL: Quality of life
